# Clinical characteristics of 172 children and adolescents with body dysmorphic disorder

**DOI:** 10.1007/s00787-020-01677-3

**Published:** 2020-11-09

**Authors:** Daniel Rautio, Amita Jassi, Georgina Krebs, Per Andrén, Benedetta Monzani, Martina Gumpert, Angela Lewis, Lauren Peile, Laura Sevilla-Cermeño, Markus Jansson-Fröjmark, Tobias Lundgren, Maria Hillborg, Maria Silverberg-Morse, Bruce Clark, Lorena Fernández de la Cruz, David Mataix-Cols

**Affiliations:** 1 Department of Clinical Neuroscience, Centre for Psychiatry Research, Karolinska Institutet, Gävlegatan 22 (Entré B), floor 8, 113 30 Stockholm, Sweden; 2 Stockholm Health Care Services, Region Stockholm, Stockholm, Sweden; 3 National and Specialist OCD, BDD, and Related Disorders Clinic for Young People, South London and Maudsley NHS Foundation Trust, London, UK; 4 Social, Genetic and Developmental Psychiatry Centre, Institute of Psychiatry, Psychology & Neuroscience, King’s College London, De Crespigny Park, London, UK; 5 Departamento de Medicina y Especialidades Médicas, Universidad de Alcalá, Madrid, Spain

**Keywords:** Body dysmorphic disorder, Dysmorphophobia, Children, Adolescents

## Abstract

Body dysmorphic disorder (BDD) often starts in childhood, with most cases developing symptoms before age 18. Yet, BDD research has primarily focused on adults. We report the clinical characteristics of the world’s largest cohort of carefully diagnosed youths with BDD and focus on previously unexplored sex and age differences. We systematically collected clinical data from 172 young people with BDD consecutively referred to 2 specialist pediatric obsessive–compulsive and related disorders outpatient clinics in Stockholm, Sweden and in London, England. A series of clinician-, self-, and parent-reported measures were administered. The cohort consisted of 136 girls, 32 boys, and 4 transgender individuals (age range 10–19 years). The mean severity of BDD symptoms was in the moderate to severe range, with more than one third presenting with severe symptoms and more than half showing poor or absent insight/delusional beliefs. We observed high rates of current psychiatric comorbidity (71.5%), past or current self-harm (52.1%), suicide attempts (11.0%), current desire for cosmetic procedures (53.7%), and complete school dropout (32.4%). Compared to boys, girls had significantly more severe self-reported BDD symptoms, depression, suicidal thoughts, and self-harm. Compared to the younger participants (14 or younger), older participants had significantly more severe compulsions and were more likely to report a desire for conducting cosmetic procedures. Adolescent BDD can be a severe and disabling disorder associated with significant risks and substantial functional impairment. The clinical presentation of the disorder is largely similar across sexes and age groups, indicating the importance of early detection and treatment. More research is needed specifically focusing on boys and pre-pubertal individuals with BDD.

**Electronic supplementary material:**

The online version of this article (10.1007/s00787-020-01677-3) contains supplementary material, which is available to authorized users.

## Introduction

Body dysmorphic disorder (BDD) is a chronic psychiatric condition characterized by a preoccupation with perceived defects or flaws in physical appearance that are not visible or appear slight to others. This preoccupation leads to significant distress and impairment, time-consuming repetitive behaviors (i.e., mirror checking, camouflaging), and marked avoidance [1]. The estimated prevalence of the disorder is approximately 2% in community samples of both adolescents and adults [2–4].

BDD usually emerges during adolescence, with a retrospectively reported mean age of onset around 16 years [5–7]. Adults who self-report an early-onset of their BDD (i.e., at age 17 or younger), compared to those with a later onset of the disorder, are thought to be more severe, more likely to have attempted suicide, and more likely to have psychiatric comorbidities [5]. However, only a handful of case series and case reports [8–11] and 3 modestly sized clinical studies (samples ranging from 30 to 36 individuals) have systematically examined the clinical characteristics of young people with BDD [6, 12, 13]. Collectively, these studies described high levels of psychiatric comorbidity, poor insight, impaired educational and social function, and high rates of seeking or receiving cosmetic or surgical treatment in an attempt to modify different aspects of their appearance.

Although these previous studies are informative, there is a need for studies in larger samples of children and adolescents with BDD including broader inclusion criteria to obtain a fuller clinical picture of the condition. For example, previous youth cohorts have been too small to examine potential differences between boys and girls with BDD. Specifically, there is an important gap in knowledge about boys with BDD, as previous cohorts were predominantly female, including only a handful of boys each [6, 12, 13]. Similarly, little is known about the developmental aspects of BDD: do younger individuals differ from late-adolescents in the severity of their symptoms, comorbidities or risky behaviors? The identification of potential differences between younger and older adolescents with BDD could help inform intervention strategies at each developmental stage.

In this naturalistic study, we report on the demographic and clinical characteristics of a uniquely large clinical sample of 172 well-characterized youth with BDD consecutively seen in specialist clinics in Stockholm, Sweden, and in London, England. In particular, we focus on potential sex and age differences, risk behaviors, and global functioning, including school attendance.

## Methods

### Setting and participants

The study was approved by the Regional Ethical Review Board in Stockholm and by the South London and Maudsley Child and Adolescent Mental Health Service Audit Committee. In the Stockholm site, informed consent was required and provided by all patients and their parents/legal guardians. In the London site, informed consent was not required because the study was part of an audit of routinely collected clinical data.

Participants were 172 children and adolescents (136 girls, 32 boys, and 4 transgender individuals: 3 assigned male at birth and identifying as female and one assigned female at birth and identifying as male) meeting Diagnostic and Statistical Manual of Mental Disorders, 5^th^ edition criteria for BDD [1]. The patients were consecutively referred to one of two national and specialist pediatric obsessive–compulsive and related disorders outpatient clinics, namely the OCD and Related Disorders Clinic for Children and Adolescents in Stockholm, Sweden (*n* = 100) or the National and Specialist OCD, BDD, and Related Disorders Clinic for Young People at the Maudsley Hospital in London, England (*n* = 72), between January 2015 and January 2020. Besides meeting diagnostic criteria for BDD and consenting to be included in the study (Stockholm site only), no other inclusion or exclusion criteria were applied. The Stockholm clinic accepts all referrals from patients diagnosed with (or suspected to have) BDD from the Stockholm Region (and occasionally other parts of Sweden and the Nordic countries), regardless of the level of symptom severity. By contrast, most patients referred to the London clinic tend to be complex cases that have often already received care in regular child and adolescent mental health services (CAMHS) before being referred to the specialist team.

Patients from both sites underwent similar assessment procedures, consisting of a 3-hour assessment by a multidisciplinary team where they completed a series of interviews, including a full psychiatric and developmental history. In Stockholm, this included the Mini International Neuropsychiatric Interview for Children (MINI-KID) [14], supplemented with additional modules for obsessive–compulsive and related disorders. In London, this was done with the Development and Well-Being Assessment (DAWBA) [15]. Interviews were administered by experienced clinical psychologists, and assessments were also discussed in multi-disciplinary teams (child psychiatrists, psychiatric nurses) before a diagnosis of BDD was confirmed. At this initial assessment, self-reported or parent-reported socio-demographic information was also gathered. For the assessment of the BDD symptom severity, the clinician-reported Yale-Brown Obsessive–Compulsive Scale Modified for BDD–Adolescent Version (BDD-YBOCS-A) [16] was administered. Additionally, other self-reported and parent-reported measures covering a range of psychiatric symptoms were collected (see “Measures” section).

In addition to the standardized measures, clinicians also asked participants about their main body areas of concern. At the Stockholm clinic, clinicians used a standardized checklist of 28 different parts of the body where the patient had to select those that they were concerned about. At the London clinic, clinicians used open-ended questions aided by a drawing of the whole body, which was used to assist patients in identifying the location of their preoccupations. Because of the different methods used to collect information about body areas of concern, these results will be reported separately by site. Detailed data on school attendance and current desire for and/or whether they had undergone any cosmetic/surgical procedure related to their BDD concerns prior to their assessment were also collected at both sites. Finally, clinicians also enquired about past or current self-harm, suicidal thoughts, and/or suicide attempts by directly asking the young person (as parents often underestimate suicidality) [17]. This information was obtained using semi-structured interviews. In the Stockholm clinic, we used the suicidality items on the MINI-KID [14] (for suicidal ideation: “Have you considered killing yourself?”; for self-harm: “Have you intentionally harmed yourself without intending to kill yourself?”; and for suicide attempts: “Have you tried to commit suicide (kill yourself)?”). In the London clinic, we used the DAWBA [15] (for suicidal ideation: “Did you think about harming yourself or killing yourself?”; for self-harm: “Have you tried to harm or hurt yourself?”; and for suicide attempts: “Did you try to harm yourself or kill yourself?”). Both instruments enquire about both current and past suicidal behaviours. Additionally, in the Stockholm clinic, the patients’ medical records were also searched for notes referring to any previous suicidal behaviour. For some analyses, these variables were pooled into a composite ‘any suicidal or self-harm behavior’ variable.

### Measures

The following clinician-administered, self-reported, and parent-reported measures were administered to all participants at both sites, unless otherwise specified.

The BDD-YBOCS is a widely-used clinician-administered, semi-structured interview that measures BDD symptom severity [16] and has good psychometric properties [18]. Here, we used the adolescent version of the BDD-YBOCS (BDD-YBOCS-A), which has not been validated separately but is nearly identical to the original adult version, with only some minimal wording differences making the items more accessible to the adolescent population. Both the BDD-YBOCS and the BDD-YBOCS-A contain 12 Likert-type items ranging from 0 to 4: 5 questions on obsessions, 5 on compulsions, 1 about insight, and 1 to measure avoidance. The total BDD severity score ranges from 0 to 48. The scale also includes an ancillary checklist for the most common BDD-related behaviors (e.g., grooming, camouflaging). This checklist was only administered at the Stockholm site.

The Appearance Anxiety Inventory (AAI) is a self-reported measure that covers typical BDD cognitions and behaviors [19]. It consists of 10 items scored on a 0–4 Likert scale, yielding a total score range from 0 to 40, and includes two subscales: avoidance and threat monitoring. It has good convergent validity, high internal consistency, and correlates well with the clinician-administered BDD-YBOCS-A [19]. The cut-off suggesting high probability of clinical problems is ≥ 20 [20].

The Clinical Global Impression-Severity scale (CGI-S) is a single-item clinician-rated measure of symptom severity ranging from 1 (‘normal, not at all ill’) to 7 (‘among the most extremely ill patients’) [21]. It is often used in treatment trials [21] and has shown good concurrent validity and sensitivity to change [22]. The CGI-S was used to assess BDD symptom severity.

Self-reported depressive symptoms were assessed by means of different measures. In Stockholm, the Children’s Depression Inventory—Short Version (CDI–S), a 10-item instrument [23], was used from 2015 and was replaced in 2018 with the Short Mood and Feeling Questionnaire (SMFQ-C), a 13-item measure [24]. During the whole time period, the 13-item parent-reported version of the instrument (SMFQ-P) was also included in the assessment battery [24]. In the London site, the 33-item Mood and Feeling Questionnaire (MFQ-C) [25] was used throughout the whole inclusion period. All used measures of depressive symptoms have shown good psychometric properties [23–26]. The suggested cut-offs that indicate clinically significant depressive symptoms for these measures are ≥ 3 for the CDI-S [23], ≥ 12 SMFQ-C and the SMFQ-P [26], and ≥ 28 for the MFQ-C [25].

The Children´s Global Assessment Scale (CGAS) is a clinician-rated measure of the global functioning of a young person during the last month. Scores range from 1 (more disabled) to 100 (best functioning). The CGAS has good psychometric properties, with high reliability as well as discriminant and concurrent validity [27].

The Work and Social Adjustment Scale – Youth (WSAS-Y) and Parent (WSAS-P) versions are short, self-reported instruments assessing functional impairment in five areas: school/work, daily situations, social activities, leisure activities, and relationships [28]. The WSAS-Y/P are based on the original WSAS for adults [29] and retain the excellent psychometric properties of the original scale, with high internal consistency, good convergent and divergent validity, and sensitivity to change [28]. The WSAS-Y/P were only administered in the Stockholm site.

### Statistical analyses

Stata 15.1 (StataCorp LLC) was used to summarize and analyze the data. Differences by sex, age at assessment, and site were explored. Student’s t-tests were used for between-group comparisons of continuous variables and Chi-squared tests for categorical variables. Statistical significance was set at *p* < 0.05. Sample sizes varied for some of the analyses as a result of missing data.

## Results

### Demographic and clinical characteristics

**Fig. 1:**
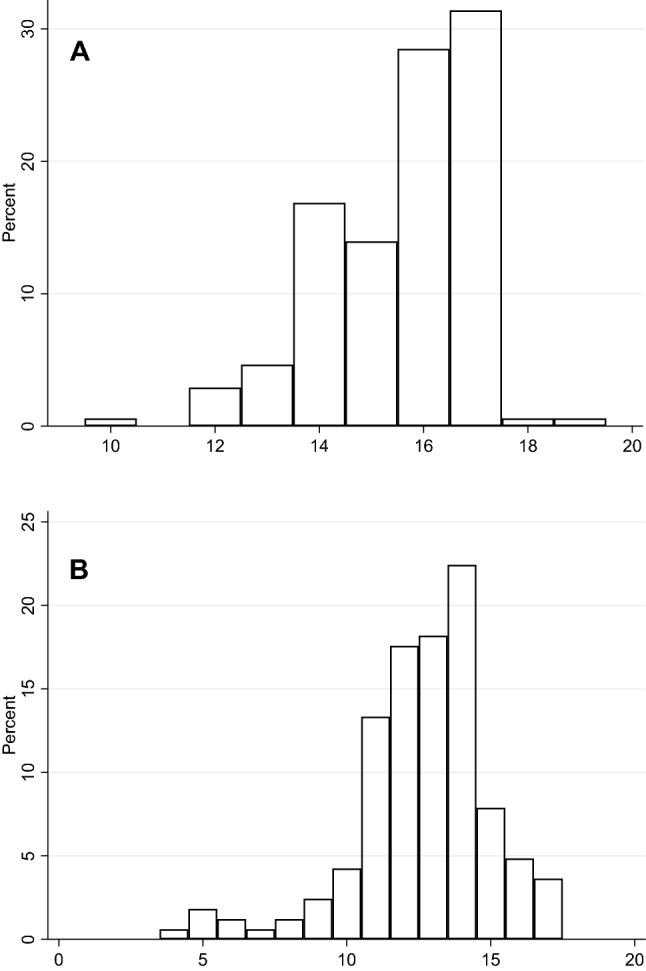
Histograms depicting the distribution of participants’ age at assessment (**a**, *N* = 172) and self-reported age at symptom onset (**b**, *N* = 165)

Demographic and clinical variables for the combined sample of 172 patients are listed in Table 1. The majority of the sample was composed of girls (*n* = 136, 79.1%). The mean age at intake was 15.6 years (SD = 1.5, range = 10–19), and the self-reported mean age of onset of BDD was 12.6 (SD = 2.4, range = 4–17). Figure 1 depicts the distribution of the participants’ age at the initial assessment (Panel A) and the self-reported age of symptom onset (Panel B).

**Table 1:** Demographic and clinical characteristics of a sample of adolescents with body dysmorphic disorder, by sex (*N* = 172)

Ages (*n*)	Combined^a^ (*N* = 172)		Boys (*n* = 32)		Girls (*n* = 136)		Statistics	
	Mean	SD	Mean	SD	Mean	SD	*t*	*p*
Age at assessment (172)	15.6	1.5	15.5	1.4	15.6	1.5	0.21	0.834
Age of BDD onset (165)	12.6	2.4	12.9	2.4	12.6	2.4	− 0.72	0.473

Clinical characteristics^b^ (*n*)	*N*	%	*N*	%	*N*	%	*χ*^2^	*p*
Any comorbid psychiatric disorder (172)	123	71.5	19	59.4	100	73.5	2.51	0.113
Mood disorders^c^	79	46.5	12	38.7	65	48.2	0.90	0.342
Anxiety disorders^d^	14	8.1	1	3.1	12	8.8	1.18	0.278
Social phobia	18	10.5	2	6.3	16	11.8	0.36	0.824
Obsessive–compulsive disorder	6	3.5	0	0	5	3.7	1.21	0.271
ADHD	17	9.9	5	15.6	11	8.1	1.71	0.191
Autism spectrum disorder	27	15.7	7	21.9	18	13.2	1.53	0.217
Eating disorders	18	10.5	3	9.4	15	11.0	0.07	0.785
Family history of OCRD (in 1st and 2nd degree relatives) (163)	50	30.7	9	30.0	40	30.8	0.01	0.934
Previous CBT for BDD (170)	23	13.5	2	6.3	21	15.6	1.89	0.170
Previous SSRI^e^ (71)	26	36.6	4	21.1	22	42.3	2.71	0.100
On pharmacological treatment (172)	94	54.7	19	59.4	71	52.2	0.54	0.464
Selective serotonin reuptake inhibitors	74	43.0	18	56.3	55	40.4	2.63	0.105
Antipsychotics	14	8.1	2	6.3	12	8.8	0.22	0.636
Antihistamines	14	8.1	0	0	12	8.8	3.04	0.081
Melatonin	19	11.1	1	3.1	16	11.8	2.13	0.145
ADHD medication	9	5.2	2	6.3	6	4.4	0.19	0.660
Poor or absent insight/delusional beliefs^f^ (162)	84	51.9	15	48.4	69	54.3	0.35	0.552
Desire for cosmetic procedure (149)	80	53.7	13	50.0	66	55.5	0.26	0.612
Conducted a cosmetic procedure (143)	14	9.8	2	8.0	12	10.5	0.14	0.704
Any suicidal or self-harm behavior (145)	98	67.6	10	40.0	85	73.3	10.36	0.001*
Past or current suicide thoughts (145)	85	58.6	10	40.0	72	62.1	4.12	0.042*
Past or current self-harm (144)	75	52.1	5	20.0	67	58.3	12.03	0.001**
History of suicide attempts (145)	16	11.0	2	8.0	13	11.2	0.22	0.637
School attendance (170)							0.05	0.977
Full attendance	53	31.2	10	32.3	41	30.4	–	–
Partial attendance	62	36.5	11	35.5	50	37.0	–	–
No attendance	55	32.4	10	32.3	44	32.6	–	–

*ADHD* attention-deficit/hyperactivity disorder, *BDD* body dysmorphic disorder, *CBT* cognitive behavior therapy, *OCRD* obsessive–compulsive and related disorders, *SD* standard deviation

*Significant at 0.05; **significant at 0.01

^a^32 boys, 136 girls, and 4 transgender individuals; ^b^ current, unless otherwise specified; ^c^ includes major depressive disorder and dysthymia; ^d^ includes specific phobia, panic disorder or anxiety disorders not otherwise specified (social phobia is reported separately); ^e^ only data from the London site; ^f^ defined as 3 or 4 on the insight item of the BDD-YBOCS-A

A total of 123 participants (71.5%) met diagnostic criteria for at least one additional psychiatric disorder, as captured by the MINI-KID or the DAWBA. The most common comorbidities were mood disorders (major depressive disorder or dysthymia, *n* = 79, 46.5%) and anxiety disorders (including social phobia, *n* = 18, 10.5%, and other anxiety disorders such as specific phobia, panic disorder or anxiety disorders not otherwise specified, *n* = 14; 8.1%). Almost one third of the participants (*n* = 50, 30.7%) had a family history of other obsessive–compulsive and related disorders. The majority of the participants (*n* = 94, 54.7%) were on psychotropic medication at the time of assessment, the most common being selective serotonin reuptake inhibitors (SSRI) (*n* = 74; 43.0%), whereas only 23 (13.5%) participants had received previous cognitive behavior therapy (CBT) for BDD.

A total of 98 (67.6%) adolescents reported suicidal or self-harm behaviors. Specifically, 16 (11.0%) patients had a history of attempted suicide, 85 (58.6%) reported past or current suicidal ideation, and 75 (52.1%) reported past or current self-harm, mainly cutting behaviors.

About one third of the participants (*n* = 53, 31.2%) had full school attendance, whereas the rest had partial attendance (*n* = 62, 36.5%) or had dropped out of school entirely (*n* = 55, 32.4%).

### BDD symptom severity and phenomenology

The mean BDD-YBOCS-A score for the whole sample was 32.04 (SD = 5.67, range = 19–44), indicating moderate to severe BDD (Table 2). A total of 58 participants (34.7%) had severe BDD symptoms (BDD-YBOCS-A scores of 35 or over). Similarly, the mean self-reported AAI score was 27.03 (SD = 7.80), which is above the suggested cut-off for clinically significant BDD symptoms [20]. The mean CGI-S score was 4.89 (SD = 0.69), indicating that the patients were assessed as being moderately to markedly ill.

**Table 2:** Clinician-, self-, and parent-reported measures in a sample of adolescents with body dysmorphic disorder, by sex (*N* = 167)

Variable (*n*)	Combined^a^ (*N* = 167)		Boys (*n* = 32)		Girls (*n* = 131)		Statistics	
	Mean	SD	Mean	SD	Mean	SD	*t*	*p*
*BDD measures*								
BDD-YBOCS-A (167)	32.04	5.67	31.72	5.63	32.21	5.74	0.44	0.659
Obsessions (160)	13.54	2.61	13.55	2.67	13.59	2.62	0.08	0.935
Compulsions (160)	13.54	2.52	13.16	2.49	13.65	2.57	0.97	0.338
Insight (166)	2.53	0.83	2.42	0.81	2.60	0.81	1.09	0.281
Avoidance (164)	2.39	0.96	2.61	0.92	2.34	0.97	− 1.46	0.151
AAI (132)	27.03	7.80	23.28	8.79	27.93	7.42	2.44	0.020*
*Other clinical measures*								
CDI-S^b^ (52)	11.15	4.56	6.13	4.32	11.90	4.02	3.50	0.006**
SMFQ-C^b^ (37)	15.32	5.34	8.80	4.27	16.13	4.70	3.27	0.014*
SMFQ-P^b^ (92)	14.55	5.95	9.77	5.29	15.30	5.71	3.44	0.003**
MFQ-C^c^ (42)	35.02	16.60	29.15	16.91	37.66	16.05	1.53	0.140
CGI-S (164)	4.89	0.69	4.74	0.63	4.93	0.71	1.46	0.150
CGAS (164)	43.48	7.73	43.47	8.07	43.42	7.75	− 0.03	0.979
WSAS-Y^b^ (95)	21.55	6.92	18.31	7.26	22.06	6.77	1.75	0.100
School or work	6.11	1.80	6.15	1.77	6.10	1.81	− 0.11	0.917
Everyday functioning	3.05	2.47	1.85	2.12	3.24	2.48	2.16	0.045*
Social activities	5.64	1.96	5.62	2.22	5.65	1.93	0.05	0.963
Spare time	2.24	2.20	0.54	1.20	2.51	2.20	4.79	0.000**
Family activities	4.51	2.14	4.15	2.88	4.56	2.02	0.49	0.631
WSAS-P^b^ (94)	21.89	7.12	22.23	5.70	21.84	7.36	− 0.22	0.828
School or work	6.39	1.95	6.85	2.08	6.32	1.93	− 0.86	0.406
Everyday functioning	3.53	2.20	3.77	2.20	3.49	2.21	− 0.42	0.681
Social activities	5.44	2.12	5.08	2.33	5.49	2.09	0.61	0.552
Spare time	2.15	2.28	2.15	2.38	2.15	2.28	− 0.01	0.994
Family activities	4.38	2.23	4.38	1.66	4.38	2.32	0.00	0.997

*BDD* body dysmorphic disorder, *AAI* Appearance Anxiety Inventory, *BDD-YBOCS-A* Yale-Brown Obsessive–Compulsive Scale, modified for BDD–Adolescent version, *CDI-S* Children’s Depression Inventory – Short Version, *CGAS* Children´s Global Assessment Scale, *CGI-S* Clinical Global Impression – Severity, *SMFQ-C* Short Mood and Feeling Questionnaire, Child Version, *SMFQ-P* Short Mood and Feeling Questionnaire, Parent Version, *MFQ-C* Mood and Feeling Questionnaire, Child Version, *WSAS-Y* Work, Social and Adjustment Scale – Youth Version, *WSAS-P* Work, Social and Adjustment Scale – Parent Version, *SD* standard deviation

^a^32 boys, 136 girls, and 4 transgender individuals; ^b^only data from the Stockholm site; ^c^only data from the London site

*Significant at 0.05; **significant at 0.01

**Fig. 2:**
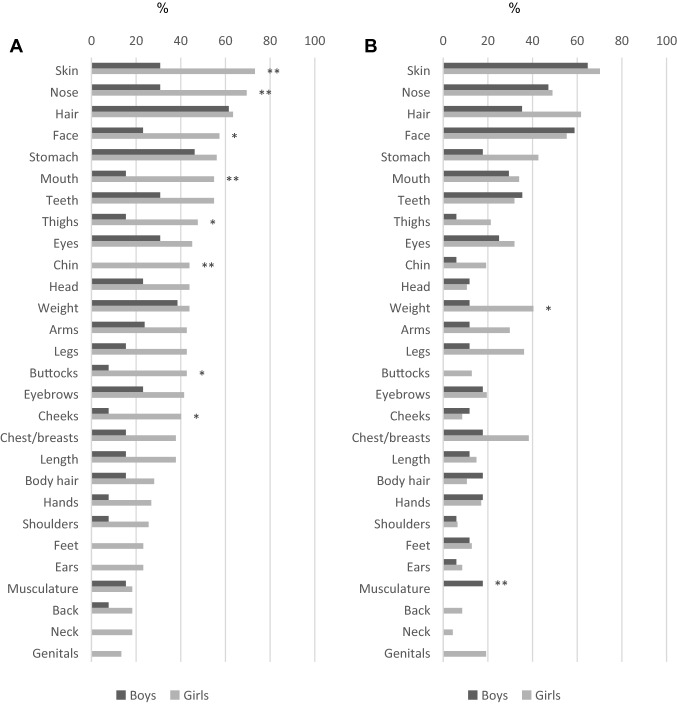
Areas of preoccupation, of a sample of adolescents with body dysmorphic disorder, by sex, in the two different sites; **a** Stockholm (*n* = 99) and **b** London (*n* = 64). *Significant at 0.05; **significant at 0.01

Most of the patients had multiple body areas of concern. The most common appearance concerns across both sites were preoccupation with the skin, nose, hair, face, and stomach (Supplementary Tables 1 and 2 and Fig. 2). The most common BDD-related behaviors, which were only reported by the Stockholm sample (Supplementary Table 3), were mirror checking (*n* = 91, 91.9%), comparing oneself with others (*n* = 90, 90.9%), camouflaging (*n* = 76, 76.8%), applying makeup (*n* = 64, 64.7%), grooming (*n* = 61, 61.6%), and reassurance seeking (*n* = 61, 61.6%).

Over half of the patients (*n* = 84, 51.9%) had poor (n = 66, 39.8%) or delusional insight (*n* = 19, 11.5%) into their perceived defects, as defined by scores of 3 or 4, respectively, on the insight item of the BDD-YBOCS-A (Table 1). Another 66 patients (39.8%) had fair insight as defined by a score of 2, 14 patients (8.4%) had good insight as defined by a score of 1 on the same item, and only 1 patient (0.6%) had excellent insight (score of 0). Furthermore, more than half of the participants reported that they desired surgery or other cosmetic procedures to correct their perceived defects (*n* = 80, 53.7%) at the time of the assessment. Fourteen individuals (9.8%) had already received some kind of minimally invasive cosmetic procedure (e.g., laser skin treatment, lip fillers) or a surgical procedure (e.g., rhinoplasty, labiaplasty, otoplasty) related to their BDD concerns at the time of the assessment.

### Depression, global severity, and functional impairment

As shown in Table 2, both the mean self- and parent-reported levels of depression were above the suggested cut-offs for clinically significant depressive symptoms. The mean CGAS score was 43.48 (SD = 7.73), suggesting a moderate functional impairment in most domains or severe impairment in one area. According to both the self- and parent-reported versions of the WSAS-Y/P, the most affected areas of functioning were school and social activities.

### Sex differences

Four individuals were transgender and were excluded from these analyses. Of note, these four participants were diagnosed with BDD on the basis on appearance concerns that were not related to their gender dysphoria.

As shown in Table 1, girls and boys did not differ significantly on most demographic or clinical variables. However, girls with BDD had significantly higher rates of any suicidal or self-harm behavior (73.3% vs. 40.0%; *χ*^2^ = 10.36, *p* = 0.001), past or current suicide thoughts (62.1% vs. 40.0%; *χ*^2^ = 4.12, *p* = 0.042), and self-harm (58.3% vs. 20.0%; *χ*^2^ = 12.03, *p* = 0.001).

**Fig. 3:**
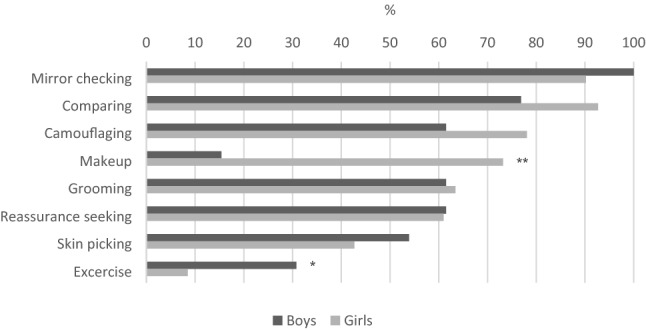
BDD-related behaviors, by sex (*N* = 99, Stockholm site only) of a sample of adolescents with body dysmorphic disorder. *Significant at 0.05; **significant at 0.01

Similarly, few sex differences were found on the clinician-, self-, and parent-reported measures. There was no difference in BDD-YBOCS-A scores (Table 2). However, girls self-reported significantly more severe BDD symptoms on the AAI, compared to boys (27.93 vs. 23.28; *t* = 2.44, *p* = 0.020) (Table 2). Although the most common body areas of preoccupation were overall similar for both sexes, some differences were found. In Stockholm, girls reported significantly more frequently preoccupation with the skin, nose, mouth, chin, face, thighs, buttocks, and cheeks, compared to boys. In London, girls reported significantly more preoccupation with weight than boys, and boys significantly more preoccupation with muscularity than girls. Furthermore, in both sites, girls reported significantly more different body areas of preoccupation than boys (11.34 vs. 5.08; *t* = 5.45, *p* < 0.001 for Stockholm; and 7.15 vs. 5.06; *t* = 2.29, *p* = 0.026 for London) (Supplementary Tables 1 and 2 and Fig. 2). Regarding BDD-related behaviors, girls reported using makeup significantly more often (73.2% vs. 15.4%; *χ*^2^ = 16.53, *p* < 0.001), while boys reported excessive training significantly more often (30.8% vs. 8.5%; *χ*^2^ = 5.42, *p* = 0.020). There were no sex differences regarding the number of different BDD-related behaviors endorsed, which on average were around five for the combined sample (mean = 5.42; SD = 1.55) (Supplementary Table 3 and Fig. 3).

Girls self-reported significantly more severe depressive symptoms on the CDI-S (*t* = 3.50, *p* = 0.006) and the SMFQ-C (*t* = 3.27, *p* = 0.014) (but not on the MFQ-C), and more severe parent-reported depressive symptoms (*t* = 3.44, *p* = 0.003). Girls also reported significantly more impairment in leisure activities (*t* = 4.79, *p* < 0.001) and everyday functioning (*t* = 2.16, *p* = 0.045), as assessed by the WSAS-Y (Table 2).

### Age differences

Based on the observed distribution of the participants’ ages (Fig. 1), we divided the sample into two age-based subgroups: participants who were up to 14 years old (*n* = 43) and those who were 15 or older (*n* = 129). Note that these groupings are relatively arbitrary and chosen to ensure a reasonable number of participants in each group for statistical analyses. The younger and older groups did not differ significantly on the majority of clinical and demographic variables (Supplementary Tables 6 and 7). However, compared to the younger participants, older participants had slightly but significantly more severe compulsions, as measured by the BDD-YBOCS-A (13.79 vs. 12.86; *t* = 2.02, *p* = 0.047), and were significantly more likely to report a desire for conducting a cosmetic procedure (60.0% vs. 36.9%, *χ*^2^ = 6.73, *p* = 0.009).

### Site differences

Because of the different nature of the two clinics, we conducted post hoc analyses to explore potential differences between sites. Patients from Stockholm and London had similar ages at assessment and a similar age of reported BDD onset. A significantly higher percentage of boys were seen in the London clinic (26.4% vs. 13.0%; *χ*^2^ = 4.96, *p* = 0.026). Patients in both sites had similar patterns of comorbidity, although the Stockholm clinic had a significantly higher proportion of social phobia (15.0% vs. 4.2%; *χ*^2^ = 5.24, *p* = 0.022) and attention-deficit/hyperactivity disorder (16.0% vs. 1.4%; *χ*^2^ = 10.03, *p* = 0.002) diagnoses. Medication regimes were also slightly different across sites, with the Stockholm patients receiving significantly more antihistamines (14.0% vs. 0%; *χ*^2^ = 10.97, *p* = 0.001), melatonin (17.0% vs. 2.8%; *χ*^2^ = 8.62, *p* = 0.003), and attention-deficit/hyperactivity disorder medication (9.0% vs. 0%; *χ*^2^ = 6.84, *p* = 0.009), and the London patients receiving significantly more SSRI medication (56.9% vs. 33.0%; *χ*^2^ = 9.79, *p* = 0.002). Significantly more patients in London had completely dropped out of school, compared to the Stockholm patients (45.1% vs. 23.2%; *χ*^2^ = 9.01, *p* = 0.003).

The London sample reported significantly more severe BDD symptoms as assessed by the BDD-YBOCS-A, but not with the self-reported AAI. The London patients also reported significantly more desire for cosmetic procedures (74.0% vs. 43.4%; *χ*^2^ = 12.48, *p* < 0.001) and more history of suicide attempts (24.4% vs. 5.0%; *χ*^2^ = 11.95, *p* < 0.001). Finally, the London sample also showed significantly more severe CGI-S and CGAS scores, while results for the rest of measures were similar across sites. Further details by site are reported in Supplementary Tables 4 and 5.

## Discussion

This study reported on the demographic and clinical characteristics of the largest cohort to date of well-characterized children and adolescents with BDD referred to two specialist CAMHS clinics. Overall, the results replicate and extend previous work with much smaller samples [6, 12, 13, 30] and provide new insights into sex and age differences in youths with BDD.

The mean age at presentation was about 16 years, while the mean age of onset of the symptoms was around 13, which is earlier than the age of onset reported in retrospective reports of adults with BDD [5–7]. Interestingly, the mean age of onset ranged from 4 to 17 years, virtually identical to the range 5 to 17 reported by Albertini and Phillips [12] in a previous study of 33 adolescent cases. These figures confirm that, while BDD is typically a disorder of adolescence, pre-pubertal onset is also possible. The age results also suggest that there is a delay of approximately 3 years between symptom onset and referral to specialist BDD services, a finding that has also been reported in other adolescent samples, with similar mean age at presentation (14.9–17.8) and mean age of onset (11.8–13.5) [6, 12, 13]. Many of the patients had been in previous contact with CAMHS and had received previous medication and/or CBT for their symptoms, but earlier detection of BDD in CAMHS and referral to specialist services should be prioritized in light of the adverse consequences associated with the disorder.

The participants in our study displayed many of the typical characteristics of BDD previously described in adults and young people with the disorder [6, 7, 12, 30]. Psychiatric comorbidity was the norm, with mood and anxiety disorders being the most common conditions, present in 47% and 19% of the sample, respectively. These percentages were lower than those reported in previous studies focused on adolescent BDD, where over 80% had comorbid mood disorders and around two thirds had comorbid anxiety disorders [6, 12]. On average, symptom severity was moderate to severe, but a substantial proportion of patients (34.7%) had symptoms in the severe range. Similar to previous studies on adolescent samples [6, 12, 30], participants reported typical preoccupations with multiple areas of their appearance (mainly skin, hair, nose, stomach, and face) and engaged in typical neutralizing strategies such as mirror checking, comparing, camouflaging, and reassurance seeking.

Approximately half of the patients were classed as having poor or absent insight/delusional beliefs into their preoccupations, in line with previous reports in adolescent BDD, which classified 50 to 60% of their samples as delusional [6, 12, 13]. A similar proportion of patients reported wanting cosmetic or surgical procedures, and nearly 10% had already undergone such procedures at the time of the assessment. Nearly 60% of the sample reported suicidal ideation, 11% had previously attempted suicide, and about half of the sample reported past or current self-harming behaviors, primarily cutting. Finally, the global functioning and the social and academic performance of the sample was also substantially impaired, with a third of the patients having dropped out of school entirely.

Overall, the results confirm those from previous studies with considerably smaller samples [6, 12, 13, 30] and indicate that pediatric BDD is a serious psychiatric condition causing a severe impact on global functioning. However, some differences are worth noting. The rates of suicidal ideation were lower in our sample (57% vs. 67–81% in previous samples) [6, 12], as were the rates of suicide attempts (11% vs. 21–44% in previous adolescent samples) [6, 12, 13]. This cannot simply be explained by different levels of BDD symptom severity (BDD-YBOCS-A scores of 32.0 vs. 30.6–37.7 in previous samples) [6, 12, 13]. A recent meta-analysis of suicidal behavior in BDD, which was primarily based on adult data, reported the weighted pooled rate of suicide attempts to be 24% [31]. The reasons for the lower rate of suicide attempts in our sample are unclear but could potentially be attributed to different approaches to the assessment of suicidality. We employed semi-structured instruments and openly asked the adolescents about suicidal behaviors but several studies have shown that young people are often reluctant to disclose suicidality to parents or health professionals [32, 33]. Thus, a multi-informant approach to ascertaining suicidality seems important in this group. Another possibility is that, as BDD becomes increasingly recognized and detected and treated in CAMHS, less complex cases are likely to be referred to specialist services like ours.

Compared to previous pediatric BDD samples where 36–47% had received surgical or medical treatment for their BDD [6, 12], fewer patients (10%) in our sample had conducted cosmetic procedures prior to their initial assessment. In terms of functional impairment, the patients in our sample were as impaired and, in some respects, even more impaired than in previous studies. A third of the combined sample, and nearly half of the London sample, had dropped out of school, compared to previously reported proportions ranging from 18 to 36.7% [6, 12, 13]. However, we did not systematically collect the specific reasons behind the school dropouts, which may have been related not only to the BDD (the primary diagnosis) but also to the accompanying comorbidities. It may be important for future studies to assess the specific reasons leading to school absence and dropouts to get further insight into this important outcome.

Previous studies of adult BDD samples indicated that the disorder is only slightly more common in women than in men [2, 34, 35], whereas our pediatric sample was largely female (81%), also in line with previous studies of youths with BDD [6, 12, 13]. The modest sample sizes of these previous investigations in youth (3, 4, and 7 boys, respectively) precluded the exploration of potential sex differences in the clinical characteristics of youth with BDD. Our sample of 32 boys represents the largest ever studied. As in adult BDD studies [36–38], we found more similarities than differences between boys and girls. However, girls with BDD self-reported more severe BDD symptoms and, as previously reported in adults [38], a greater number of different appearance preoccupations. Contrary to studies in adults [36–38], where preoccupation with genitalia has been reported to be more common among men, there were no significant differences in preoccupation with genitals between boys and girls in our sample. No boys endorsed this preoccupation in any of the samples (vs. 11 girls in Stockholm and 8 in London). It is possible that boys find it particularly difficult to disclose these symptoms. Preoccupation with muscularity was, in line with the adult studies [36–38], more common among boys in the London sample, but not among the patients from Stockholm. As previously reported in adults [38], boys were more involved in excessive training and female patients used more makeup to conceal their perceived flaws. Girls also reported more self-reported symptoms of depression (but did not have a higher prevalence of clinician-assessed mood disorders) and had higher rates of suicide thoughts and self-harm, but not suicide attempts, although numbers for this variable were small. This pattern matches the overall sex differences in the suicidality literature [39]. Whereas previous studies of adults with BDD [36–38] found sex differences in comorbidities, with eating disorder and bipolar disorder being more common in women and substance abuse being more common in men, in our sample there were no differences in comorbidities between boys and girls. Furthermore, previous studies have also reported more severe functional impairment among men due to their BDD [36, 38]. However, the impact in everyday function due to BDD was similar between the boys and girls in our sample.

Four of our participants self-identified as being transgender. To our knowledge, there are no previous reports of transgender young people with BDD. A carefully differential diagnosis is needed to differentiate body image concerns that are strictly related to gender dysphoria and those that are typical of BDD and hence warrant a BDD diagnosis [1, 40].

Very little is known about the clinical presentation of BDD across different age groups. We had few children in our sample and therefore our results should be interpreted cautiously. Compared to the younger participants (14 or younger), older participants had more severe compulsions and were more likely to report a desire for conducting a cosmetic procedure. However, there were more similarities than differences, suggesting that BDD presents similarly across the studied age range and that early detection is important. Additionally, individuals in our sample reported onset of BDD symptom at as young as 4 years of age, which highlights the need to further study and understand the clinical characteristics of pre-pubertal individuals with BDD.

This study’s results are useful to inform clinical practice. Based on our findings, it is important to perform structured suicide risk assessments in this group, ideally using multiple informants. Because of the high risk of school failure, it may also be important to screen for BDD among young people with school refusal problems. Missing school can have devastating consequences for a young person, markedly reducing opportunities for higher education and employment, further increasing the impairment caused by BDD. Furthermore, since poor insight and a desire for cosmetic treatment are common in BDD, collaborations with dermatologists, beauty clinics, and plastic surgeons are needed to help detect patients with BDD and minimize unnecessary procedures. The current evidence, although scarce, suggests that cosmetic interventions in individuals with BDD are typically associated with poor outcomes and, therefore, the wish for cosmetic and surgical interventions in children and adolescents should be handled with the greatest caution and generally discouraged [41]. Lastly, considering the low proportion of male patients, it is possible that the prevalence of BDD in girls is higher, as suggested in a previous population based-study of BDD symptoms in adolescents [3]. However, another possibility is that BDD symptoms are more difficult to detect in boys and, therefore, it is important to be extra thorough in screening for the disorder in boys. Socio-cultural factors may also play a role whereby it may be less socially acceptable for boys to talk about appearance-related issues, leading to less willingness to seek help in this group and/or potential referral biases.

The main strength of this study was the large and diverse sample, consecutively collected without exclusion criteria from two different European countries. Due to the large sample, we were able to study sex differences in youth, which has not been done before, and we were also able to replicate the findings from previous studies with smaller sample sizes. Another strength was that we used gold standard measures and collected information on a wide range of clinically relevant aspects. An important limitation to consider is that all patients came from specialist settings, which may have an impact on the generalizability of the results. We pooled data from two different sites, which may have increased the variability in the data, adding a potential bias in reference to variation in measurement and clinical practices. The two sites also had some differences on the measures used and the methods of collection of these measures, although they were largely overlapping. The interrater reliability for clinician-rated measures was not established across sites. The patients on both sites were remarkably similar but there were also some differences, which can be explained by the different referral pathways in each country. In general, the London patients were more severe, had higher suicidality, and more school failure. Despite the large number of comparisons, alpha levels were not adjusted, potentially resulting in a risk of type I error. However, given the descriptive and exploratory nature of the study, and the non-independence of the variables under study, we decided not to apply multiple corrections. Since this was a naturalistic study using data collected in actual clinical settings for a period of 5 years, we had some expected data loss in different parts of the process. Finally, we did not include general population controls or other clinical groups, which would have been helpful to put the results in a wider context.

## Conclusions

Child and adolescent BDD can be a severe and disabling condition associated with several important risks, including suicide risk, self-harm behaviors, desire for unhelpful cosmetic procedures, and school refusal. The clinical presentation of the disorder is largely similar between boys and girls, although girls tend to self-report more severe BDD and depression symptoms and present with higher self-harm rates. BDD appears to be equally serious, impairing, and risky in children and younger adolescents, compared to older adolescents. Our results highlight the importance of early detection and treatment, as well as the need for more research on boys and pre-pubertal individuals with BDD.

## Electronic supplementary material

Below is the link to the electronic supplementary material.

Supplementary file1 (DOCX 47 KB)
