# Supplementary material for: Clinical characteristics of 172 children and adolescents with body dysmorphic disorder
Source: Eur Child Adolesc Psychiatry. 2020 Nov 9;31(1):133–44. doi: 10.1007/s00787-020-01677-3 (PMC8817062; doi:10.1007/s00787-020-01677-3)
Supplement: Supplementary file 1 — Supplementary file1 (DOCX 47 KB) [file 787_2020_1677_MOESM1_ESM.docx]

**SUPPLEMENTARY MATERIAL**

**Supplementary Table 1** Appearance concerns in a sample of adolescents with body dysmorphic disorder, by sex, Stockholm site (*N*=99)

|  | **Combined**^a^  **(*N*=99)** | | **Boys**  **(*n* = 13)** | | **Girls**  **(*n* = 82)** | | **Statistics** | |
| --- | --- | --- | --- | --- | --- | --- | --- | --- |
| **Variable** | **Mean** | **SD** | **Mean** | **SD** | **Mean** | **SD** | ***t*** | ***p*** |
| Number of concerns | 10.45 | 6.47 | 5.08 | 3.25 | 11.34 | 6.46 | 5.45 | 0.000** |
|  | ***N*** | **%** | ***N*** | **%** | ***N*** | **%** | ***χ^2^*** | ***P*** |
| **Body area** |  |  |  |  |  |  |  |  |
| Skin | 67 | 67.7 | 4 | 30.8 | 60 | 73.2 | 9.18 | 0.002** |
| Nose | 63 | 63.6 | 4 | 30.8 | 57 | 69.5 | 7.33 | 0.007** |
| Hair | 63 | 63.6 | 8 | 61.5 | 52 | 63.4 | 0.02 | 0.896 |
| Face | 52 | 52.5 | 3 | 23.1 | 47 | 57.3 | 5.28 | 0.022* |
| Stomach | 53 | 53.4 | 6 | 46.2 | 46 | 56.1 | 0.45 | 0.503 |
| Mouth | 48 | 48.5 | 2 | 15.4 | 45 | 54.9 | 7.00 | 0.008** |
| Teeth | 51 | 51.5 | 4 | 30.8 | 45 | 54.9 | 2.61 | 0.106 |
| Thighs | 42 | 42.4 | 2 | 15.4 | 39 | 47.6 | 4.74 | 0.030* |
| Eyes | 43 | 43.4 | 4 | 30.8 | 37 | 45.1 | 0.94 | 0.332 |
| Chin | 37 | 37.4 | 0 | 0 | 36 | 43.9 | 9.19 | 0.002** |
| Head | 42 | 42.4 | 3 | 23.1 | 36 | 43.9 | 2.01 | 0.156 |
| Weight | 42 | 42.4 | 5 | 38.5 | 36 | 43.9 | 0.14 | 0.713 |
| Arms | 39 | 39.4 | 3 | 23.8 | 35 | 42.7 | 1.80 | 0.180 |
| Legs | 39 | 39.4 | 2 | 15.4 | 35 | 42.7 | 3.52 | 0.061 |
| Buttocks | 37 | 37.4 | 1 | 7.7 | 35 | 42.7 | 5.84 | 0.016* |
| Eyebrows | 38 | 38.4 | 3 | 23.1 | 34 | 41.5 | 1.60 | 0.207 |
| Cheeks | 35 | 35.4 | 1 | 7.7 | 33 | 40.2 | 5.17 | 0.023* |
| Chest/breasts | 34 | 34.4 | 2 | 15.4 | 31 | 37.8 | 2.49 | 0.115 |
| Length | 34 | 34.4 | 2 | 15.4 | 31 | 37.8 | 2.49 | 0.115 |
| Body hair | 26 | 26.3 | 2 | 15.4 | 23 | 28.1 | 0.93 | 0.335 |
| Hands | 25 | 25.3 | 1 | 7.7 | 22 | 26.8 | 2.24 | 0.135 |
| Shoulders | 23 | 23.2 | 1 | 7.7 | 21 | 25.6 | 2.02 | 0.155 |
| Feet | 20 | 20.2 | 0 | 0 | 19 | 23.2 | 3.77 | 0.052 |
| Ears | 19 | 19.2 | 0 | 0 | 19 | 23.2 | 3.77 | 0.052 |
| Musculature | 18 | 18.2 | 2 | 15.4 | 15 | 18.3 | 0.06 | 0.799 |
| Back | 18 | 18.2 | 1 | 7.7 | 15 | 18.3 | 0.90 | 0.343 |
| Neck | 15 | 15.2 | 0 | 0 | 15 | 18.3 | 2.82 | 0.093 |
| Genitals | 12 | 12.1 | 0 | 0 | 11 | 13.4 | 1.97 | 0.160 |
| * Significant at 0.05; ** significant at 0.01.  *Note:* ^a^ Includes 13 boys, 82 girls, and 4 transgender individuals.  *Abbreviations:* SD, standard deviation. | | | | | | | | |

**Supplementary Table 2** Appearance concerns in a sample of adolescents with body dysmorphic disorder, by sex, London site (*N*=64)

|  | **Combined**  **(*N*=64)** | | **Boys**  **(*n*=17)** | | **Girls**  **(*n*=47)** | | **Statistics** | |
| --- | --- | --- | --- | --- | --- | --- | --- | --- |
| **Variable** | **Mean** | **SD** | **Mean** | **SD** | **Mean** | **SD** | ***t*** | ***P*** |
| Number of concerns | 6.59 | 4.18 | 5.06 | 2.61 | 7.15 | 4.51 | 2.29 | 0.026* |
|  | ***N*** | **%** | ***N*** | **%** | ***N*** | **%** | ***χ^2^*** | ***P*** |
| **Body area** |  |  |  |  |  |  |  |  |
| Skin | 44 | 68.7 | 33 | 64.7 | 11 | 70.2 | 0.18 | 0.675 |
| Nose | 31 | 48.4 | 8 | 47.1 | 23 | 48.9 | 0.02 | 0.894 |
| Hair | 35 | 54.7 | 6 | 35.3 | 29 | 61.7 | 3.51 | 0.061 |
| Face | 36 | 56.3 | 10 | 58.8 | 26 | 55.3 | 0.06 | 0.803 |
| Stomach | 23 | 35.9 | 3 | 17.7 | 20 | 42.6 | 3.36 | 0.067 |
| Mouth | 21 | 32.8 | 5 | 29.4 | 16 | 34.0 | 0.12 | 0.727 |
| Teeth | 21 | 32.8 | 6 | 35.4 | 15 | 31.9 | 0.06 | 0.799 |
| Thighs | 11 | 17.2 | 1 | 5.9 | 10 | 21.3 | 2.08 | 0.149 |
| Eyes | 19 | 30.2 | 4 | 25.0 | 15 | 31.9 | 0.27 | 0.603 |
| Chin | 10 | 15.6 | 1 | 5.9 | 9 | 19.2 | 1.67 | 0.197 |
| Head | 7 | 10.9 | 2 | 11.8 | 5 | 10.6 | 0.02 | 0.899 |
| Weight | 21 | 32.8 | 2 | 11.8 | 19 | 40.4 | 4.65 | 0.031* |
| Arms | 16 | 25.0 | 2 | 11.8 | 14 | 29.8 | 2.16 | 0.141 |
| Legs | 19 | 29.7 | 2 | 11.8 | 17 | 36.2 | 3.56 | 0.059 |
| Buttocks | 6 | 9.4 | 0 | 0 | 6 | 12.8 | 2.39 | 0.122 |
| Eyebrows | 12 | 18.8 | 3 | 17.7 | 9 | 19.5 | 0.19 | 0.892 |
| Cheeks | 6 | 9.4 | 2 | 11.8 | 4 | 8.5 | 0.16 | 0.693 |
| Chest/breasts | 21 | 32.8 | 3 | 17.7 | 18 | 38.3 | 2.42 | 0.120 |
| Length | 9 | 14.1 | 2 | 11.8 | 7 | 14.9 | 0.10 | 0.750 |
| Body hair | 8 | 12.5 | 3 | 17.7 | 5 | 10.6 | 0.56 | 0.454 |
| Hands | 11 | 17.2 | 3 | 17.7 | 8 | 17.0 | 0.00 | 0.953 |
| Shoulders | 4 | 6.3 | 1 | 5.9 | 3 | 6.4 | 0.01 | 0.942 |
| Feet | 8 | 12.5 | 2 | 11.8 | 6 | 12.8 | 0.01 | 0.915 |
| Ears | 5 | 7.8 | 1 | 5.9 | 4 | 8.5 | 0.12 | 0.729 |
| Musculature | 3 | 4.7 | 3 | 17.7 | 0 | 0 | 8.70 | 0.003** |
| Back | 4 | 6.3 | 0 | 0 | 4 | 8.5 | 1.54 | 0.214 |
| Neck | 2 | 3.1 | 0 | 0 | 2 | 4.3 | 0.75 | 0.388 |
| Genitals | 9 | 12.1 | 0 | 0 | 9 | 19.2 | 3.79 | 0.052 |
| * Significant at 0.05; ** significant at 0.01.  *Abbreviations:* SD, standard deviation. | | | | | | | | |

**Supplementary Table 3** BDD-related behaviors in a sample of adolescents with body dysmorphic disorder, by sex (*N*=99). Only data from the Stockholm site

|  | **Combined**^a^  **(*n*=99)** | | **Boys**  **(*n*=13)** | | **Girls**  **(*n*=82)** | | **Statistics** | |
| --- | --- | --- | --- | --- | --- | --- | --- | --- |
| **Variable** | **Mean** | **SD** | **Mean** | **SD** | **Mean** | **SD** | ***t*** | ***p*** |
| Number of compulsions | 5.42 | 1.55 | 4.85 | 1.34 | 5.51 | 1.60 | 1.61 | 0.124 |
|  | ***N*** | **%** | ***N*** | **%** | ***N*** | **%** | ***χ^2^*** | ***p*** |
| **BDD-related behaviors** |  |  |  |  |  |  |  |  |
| Mirror checking | 91 | 91.9 | 13 | 100.0 | 74 | 90.2 | 1.38 | 0.239 |
| Comparing | 90 | 90.9 | 10 | 76.9 | 76 | 92.7 | 3.25 | 0.071 |
| Camouflaging | 76 | 76.8 | 8 | 61.5 | 64 | 78.1 | 1.67 | 0.197 |
| Makeup | 64 | 64.7 | 2 | 15.4 | 60 | 73.2 | 16.53 | 0.000** |
| Grooming | 61 | 61.6 | 8 | 61.5 | 52 | 63.4 | 0.02 | 0.896 |
| Reassurance seeking | 61 | 61.6 | 8 | 61.5 | 50 | 61.0 | 0.00 | 0.969 |
| Skin picking | 44 | 44.4 | 7 | 53.9 | 35 | 42.7 | 0.57 | 0.451 |
| Exercise | 11 | 11.1 | 4 | 30.8 | 7 | 8.5 | 5.42 | 0.020* |
| Other^b^ | 39 | 39.4 | 3 | 23.1 | 34 | 41.5 | 1.60 | 0.207 |
| * Significant at 0.05; ** significant at 0.01.  *Note:*  ^a^ Includes 82 girls, 13 boys and 4 transgender individuals; ^b^ e.g., searching information on cosmetic procedures on the Internet, taking pictures of oneself.  *Abbreviations:* BBD, body dysmorphic disorder; SD, standard deviation. | | | | | | | | |

**Supplementary Table 4** Demographic and clinical characteristics of a sample of adolescents with body dysmorphic disorder, by site (*N*=172)

|  | **Stockholm**  **(*n=*100)** | | **London**  **(*n=*72)** | | **Statistics** | |
| --- | --- | --- | --- | --- | --- | --- |
| **Ages** (n) | **Mean** | **SD** | **Mean** | **SD** | ***t*** | ***p*** |
| **Age at assessment** (172) | 15.4 | 1.5 | 15.8 | 1.4 | -1.46 | 0.147 |
| **Age of BDD onset** (165) | 12.7 | 1.9 | 12.5 | 3.0 | 0.54 | 0.590 |
| **Clinical characteristics**^a^ (n) | ***N*** | **%** | ***N*** | **%** | ***χ^2^*** | ***p*** |
| **Sex** (172) |  |  |  |  | 7.38 | 0.025* |
| Girls | 83 | 83.0 | 53 | 73.6 | 2.23 | 0.135 |
| Boys | 13 | 13.0 | 19 | 26.4 | 4.96 | 0.026* |
| Transgender | 4 | 4.0 | 0 | 0 | 2.95 | 0.086 |
| **Any comorbid mental disorder** (172) | 75 | 75.0 | 48 | 66.7 | 1.43 | 0.232 |
| Mood disorders^b^ | 49 | 49.0 | 30 | 42.9 | 0.62 | 0.429 |
| Anxiety disorders^c^ | 8 | 8.0 | 6 | 8.3 | 0.01 | 0.937 |
| Social phobia | 15 | 15.0 | 3 | 4.2 | 5.24 | 0.022* |
| Obsessive-compulsive disorder | 5 | 5.0 | 1 | 1.4 | 1.62 | 0.203 |
| Attention-deficit/hyperactivity disorder | 16 | 16.0 | 1 | 1.4 | 10.03 | 0.002** |
| Autism spectrum disorder | 14 | 14.0 | 13 | 18.1 | 0.52 | 0.471 |
| Eating disorders | 7 | 7.0 | 11 | 15.3 | 3.06 | 0.080 |
| **Family history (1^st^ and 2^nd^ degree relatives) of OCDRD** (163) | 30 | 30.6 | 20 | 30.8 | 0.00 | 0.983 |
| **Previous CBT for BDD** (170) | 10 | 10.2 | 13 | 18.1 | 2.19 | 0.139 |
| **On pharmacological treatment** (172) | 49 | 49.0 | 45 | 62.5 | 3.08 | 0.079 |
| Selective serotonin reuptake inhibitors | 33 | 33.0 | 41 | 56.9 | 9.79 | 0.002** |
| Antipsychotics | 5 | 5.0 | 9 | 12.5 | 3.15 | 0.076 |
| Antihistamines | 14 | 14.0 | 0 | 0 | 10.97 | 0.001** |
| Melatonin | 17 | 17.0 | 2 | 2.8 | 8.62 | 0.003** |
| ADHD medication | 9 | 9.0 | 0 | 0 | 6.84 | 0.009** |
| **Poor or absent insight/delusional beliefs^d^** (162) | 47 | 47.5 | 37 | 58.7 | 1.95 | 0.162 |
| **Desire for cosmetic procedure** (149) | 43 | 43.4 | 37 | 74.0 | 12.48 | 0.000** |
| **Conducted a cosmetic procedure** (143) | 7 | 7.1 | 7 | 15.9 | 2.69 | 0.101 |
| **Any suicidal or self-harm behavior** (145) | 67 | 67.0 | 31 | 68.9 | 0.05 | 0.822 |
| Past or current suicide thoughts (145) | 54 | 54.0 | 31 | 68.9 | 2.84 | 0.092 |
| Past or current self-harm (144) | 54 | 54.0 | 21 | 47.7 | 0.48 | 0.488 |
| History of suicide attempts (145) | 5 | 5.0 | 11 | 24.4 | 11.95 | 0.001** |
| **School attendance** (170) |  |  |  |  | 26.50 | 0.000** |
| Full attendance | 24 | 24.2 | 29 | 40.9 | 5.31 | 0.021* |
| Partial attendance | 52 | 52.5 | 10 | 14.1 | 26.37 | 0.000** |
| No attendance | 23 | 23.2 | 32 | 45.1 | 9.01 | 0.003** |
| * Significant at 0.05; ** significant at 0.01  *Note:* ^a^ Current, unless otherwise specified; ^b^ includes major depressive disorder and dysthymia; ^c^ includes specific phobia, panic disorder or anxiety disorders not otherwise specified (social phobia is reported separately); ^d^ defined as 3 or 4 on the insight item of the BDD-YBOCS-A.  *Abbreviations:* BBD, body dysmorphic disorder; ADHD, attention-deficit/hyperactivity disorder; CBT, cognitive behavior therapy; OCRD, obsessive-compulsive and related disorder; SD, standard deviation. | | | | | | |

**Supplementary Table 5** Clinician-, self-, and parent-reported measures in a sample of adolescents with body dysmorphic disorder, by site (*N*=167)

|  | **Stockholm**  **(*n*=99)** | | **London**  **(*n*=68)** | | **Statistics** | |
| --- | --- | --- | --- | --- | --- | --- |
| **Variable** (n) | **Mean** | **SD** | **Mean** | **SD** | ***t*** | ***p*** |
| **BDD measures** |  |  |  |  |  |  |
| **BDD-YBOCS-A** (167) | 30.68 | 5.11 | 34.01 | 5.89 | -3.79 | 0.000** |
| Obsessions (160) | 13.04 | 2.37 | 14.36 | 2.79 | -3.08 | 0.003** |
| Compulsions (160) | 13.10 | 2.34 | 14.26 | 2.66 | -2.81 | 0.006** |
| Insight (166) | 2.35 | 0.81 | 2.80 | 0.79 | -3.59 | 0.001** |
| Avoidance (164) | 2.15 | 0.89 | 2.75 | 0.95 | -4.07 | 0.000** |
| **AAI** (132) | 27.39 | 7.43 | 26.28 | 8.54 | 0.73 | 0.467 |
| **Other clinical measures** |  |  |  |  |  |  |
| **CGI-S** (164) | 4.78 | 0.70 | 5.06 | 0.65 | -2.67 | 0.009** |
| **CGAS** (164) | 46.0 | 6.68 | 39.63 | 7.67 | 5.47 | 0.000** |
| * Significant at 0.05; ** significant at 0.01.  *Abbreviations:* BBD, body dysmorphic disorder; AAI, Appearance Anxiety Inventory; BDD, body dysmorphic disorder; BDD-YBOCS-A, Yale-Brown Obsessive-Compulsive Scale Modified for BDD–Adolescent version; CGAS, Children´s Global Assessment Scale; CGI-S, Clinical Global Impression – Severity; SD, standard deviation. | | | | | | |

**Supplementary Table 6** Demographic and clinical characteristics of a sample of adolescents with body dysmorphic disorder, by age at assessment (*N*=172)

|  | **Age** $\boldsymbol{\leq}$**14 years old**  **(*n=*43)** | | **Age >14 years old**  **(*n=*129)** | | **Statistics** | |
| --- | --- | --- | --- | --- | --- | --- |
| **Ages (n)** | **Mean** | **SD** | **Mean** | **SD** | ***t*** | ***P*** |
| **Age of BDD onset** (165) | 11.8 | 2.2 | 12.9 | 2.4 | 2.80 | 0.007* |
| **Clinical characteristics**^a^ (n) | ***N*** | **%** | ***N*** | **%** | ***χ^2^*** | ***P*** |
| **Sex** (172) |  |  |  |  | 1.66 | 0.437 |
| Girls | 36 | 83.7 | 100 | 77.5 | - | - |
| Boys | 7 | 16.3 | 25 | 19.4 | - | - |
| Transgender | 0 | 0 | 4 | 3.1 | - | - |
| **Any comorbid mental disorder** (172) | 28 | 65.1 | 95 | 73.6 | 1.15 | 0.283 |
| Mood disorders^b^ | 18 | 42.9 | 61 | 47.7 | 0.29 | 0.588 |
| Anxiety disorders^c^ | 3 | 7.0 | 11 | 8.5 | 0.10 | 0.747 |
| Social phobia | 5 | 11.6 | 13 | 10.1 | 0.08 | 0.774 |
| Obsessive-compulsive disorder | 2 | 4.7 | 4 | 3.1 | 0.23 | 0.631 |
| Attention-deficit/hyperactivity disorder | 2 | 4.7 | 15 | 11.6 | 1.76 | 0.184 |
| Autism spectrum disorder | 5 | 11.6 | 22 | 17.1 | 0.72 | 0.397 |
| Eating disorders | 4 | 9.3 | 14 | 10.9 | 0.08 | 0.774 |
| **Family history (1^st^ and 2^nd^ degree relatives) of OCDRD** (163) | 13 | 30.2 | 37 | 30.8 | 0.01 | 0.942 |
| **Previous CBT for BDD** (170) | 3 | 7.0 | 20 | 15.8 | 2.11 | 0.146 |
| **Previous SSRI**^d^ (71) | 3 | 21.4 | 23 | 40.4 | 1.73 | 0.188 |
| **On pharmacological treatment** (172) | 19 | 44.2 | 75 | 58.1 | 2.53 | 0.111 |
| Selective serotonin reuptake inhibitors | 15 | 34.9 | 59 | 45.7 | 1.55 | 0.213 |
| Antipsychotics | 2 | 4.7 | 12 | 9.3 | 0.93 | 0.334 |
| Antihistamines | 3 | 7.0 | 11 | 8.5 | 0.10 | 0.747 |
| Melatonin | 3 | 7.0 | 16 | 12.4 | 0.97 | 0.326 |
| ADHD medication | 0 | 0 | 9 | 7.0 | 3.17 | 0.075 |
| **Poor or absent insight/delusional beliefs^e^** (162) | 23 | 54.8 | 61 | 50.8 | 0.19 | 0.661 |
| **Desire for cosmetic procedure** (149) | 14 | 35.9 | 66 | 60.0 | 6.73 | 0.009** |
| **Conducted a cosmetic procedure** (143) | 2 | 5.3 | 12 | 11.4 | 1.20 | 0.273 |
| **Any suicidal or self-harm behavior** (145) | 25 | 65.8 | 73 | 68.2 | 0.08 | 0.783 |
| Past or current suicide thoughts (145) | 19 | 50.0 | 66 | 61.7 | 1.58 | 0.209 |
| Past or current self-harm (144) | 20 | 52.6 | 55 | 51.9 | 0.01 | 0.937 |
| History of suicide attempts (145) | 5 | 13.2 | 11 | 10.3 | 0.24 | 0.627 |
| **School attendance** (170) |  |  |  |  | 3.51 | 0.173 |
| Full attendance | 15 | 34.9 | 38 | 29.9 | - | - |
| Partial attendance | 19 | 44.2 | 43 | 33.9 | - | - |
| No attendance | 9 | 20.9 | 46 | 36.2 | - | - |
| * Significant at 0.05; ** significant at 0.01  *Note:* ^a^ Current, unless otherwise specified; ^b^ includes major depressive disorder and dysthymia; ^c^ includes specific phobia, panic disorder or anxiety disorders not otherwise specified (social phobia is reported separately); ^d^ only data from the London site; ^e^ defined as 3 or 4 on the insight item of the BDD-YBOCS-A.  *Abbreviations:* BBD, body dysmorphic disorder; ADHD, attention-deficit/hyperactivity disorder; CBT, cognitive behavior therapy; OCRD, obsessive-compulsive and related disorder; SD, standard deviation. | | | | | | |

**Supplementary Table 7** Clinician-, self-, and parent-reported measures in a sample of adolescents with body dysmorphic disorder, by age at assessment (*N*=167)

|  | **Age** $\boldsymbol{\leq}$**14 years old**  **(*n=*43)** | | **Age >14 years old**  **(*n=*129)** | | **Statistics** | |
| --- | --- | --- | --- | --- | --- | --- |
| **Variable** (n) | **Mean** | **SD** | **Mean** | **SD** | ***t*** | ***p*** |
| **BDD measures** |  |  |  |  |  |  |
| **BDD-YBOCS-A** (167) | 30.67 | 6.04 | 32.50 | 5.49 | 1.74 | 0.087 |
| Obsessions (160) | 12.98 | 2.84 | 13.75 | 2.51 | 1.55 | 0.125 |
| Compulsions (160) | 12.86 | 2.60 | 13.79 | 2.46 | 2.02 | 0.047* |
| Insight (166) | 2.60 | 0.86 | 2.51 | 0.82 | -0.58 | 0.567 |
| Avoidance (164) | 2.31 | 0.98 | 2.42 | 0.95 | 0.63 | 0.534 |
| **AAI** (132) | 24.94 | 7.97 | 27.81 | 7.62 | 1.86 | 0.067 |
| **Other clinical measures** |  |  |  |  |  |  |
| **CDI-S**^b^ (52) | 11.41 | 4.33 | 11.03 | 4.73 | -0.29 | 0.773 |
| **SMFQ-C**^b^ (37) | 17.18 | 4.56 | 14.54 | 5.54 | -1.51 | 0.145 |
| **SMFQ-P**^b^ (92) | 15.10 | 5.85 | 14.30 | 6.02 | -0.61 | 0.547 |
| **MFQ-C**^c^ (42) | 21.33 | 19.90 | 37.31 | 15.12 | 1.88 | 0.109 |
| **CGI-S** (164) | 4.79 | 0.75 | 4.93 | 0.67 | 1.08 | 0.286 |
| **CGAS** (164) | 44.14 | 7.22 | 43.24 | 7.91 | -0.68 | 0.496 |
| **WSAS-Y**^b^ (95) | 20.17 | 6.45 | 22.15 | 7.08 | 1.34 | 0.187 |
| School or work | 5.79 | 1.82 | 6.24 | 1.78 | 1.12 | 0.270 |
| Everyday functioning | 2.52 | 2.43 | 3.29 | 2.47 | 1.42 | 0.163 |
| Social activities | 5.31 | 2.25 | 5.79 | 1.81 | 1.01 | 0.319 |
| Spare time | 2.52 | 2.37 | 2.12 | 2.12 | -0.77 | 0.443 |
| Family activities | 4.03 | 2.04 | 4.71 | 2.17 | 1.46 | 0.150 |
| **WSAS-P**^b^ (94) | 20.45 | 6.87 | 22.54 | 7.19 | 1.34 | 0.185 |
| School or work | 6.00 | 2.10 | 6.57 | 1.86 | 1.25 | 0.216 |
| Everyday functioning | 3.07 | 2.17 | 3.74 | 2.20 | 1.38 | 0.174 |
| Social activities | 5.07 | 2.49 | 5.60 | 1.93 | 1.02 | 0.314 |
| Spare time | 2.17 | 2.39 | 2.14 | 2.24 | -0.06 | 0.949 |
| Family activities | 4.14 | 2.13 | 4.49 | 2.29 | 0.73 | 0.470 |
| * Significant at 0.05; ** significant at 0.01.  *Note:* ^a^ Includes 32 boys, 136 girls, and 4 transgender individuals; ^b^ only data from the Stockholm site; ^c^ only data from the London site.  *Abbreviations:* BBD, body dysmorphic disorder; AAI, Appearance Anxiety Inventory; BDD-YBOCS-A, Yale-Brown Obsessive-Compulsive Scale, modified for BDD–Adolescent version; CDI-S, Children’s Depression Inventory – Short Version; CGAS, Children´s Global Assessment Scale; CGI-S, Clinical Global Impression – Severity; SMFQ-C, Short Mood and Feeling Questionnaire, Child Version; SMFQ-P, Short Mood and Feeling Questionnaire, Parent Version; MFQ-C, Mood and Feeling Questionnaire, Child Version; WSAS-Y, Work, Social and Adjustment Scale – Youth Version; WSAS-P, Work, Social and Adjustment Scale – Parent Version; SD, standard deviation. | | | | | | |
